# Recurrent Catamenial Pneumothorax Suggestive of Pleural Endometriosis

**DOI:** 10.1155/2014/756040

**Published:** 2014-09-30

**Authors:** Shawky Z. A. Badawy, Pujan Shrestha

**Affiliations:** Division of Reproductive Endocrinology and Infertility, Department of Obstetrics and Gynecology, SUNY, Upstate Medical University, Syracuse, NY, USA

## Abstract

A 42-year-old multiparous patient presented for consultation as a referral for management of recurrent catamenial pneumothorax. Evaluation by a pulmonologist failed to reveal any chest masses. She was treated for endometriosis using Danazol 800 mg daily for 6 months. Pneumothorax did not recur during treatment and during follow-up visits.

## 1. Introduction

Endometriosis is a disease process that affects various organs in women [1]. It is a serious health issue because it requires many office visits and several lines of treatment because of recurrence of the disease, and it affects work attendance.

Endometriosis is the result of the growth and proliferation of endometrial-like tissue including glands and stroma in various parts of the body. It has been reported, mostly, in the pelvic cavity. It also has been shown to affect the abdominal cavity especially the pelvic colon and the terminal ileum, cecum, and small intestines [2]. Reports of endometriosis have been published indicating that it affects cesarean section scars as well as episiotomy scars [3].

Pulmonary endometriosis is thought to be a rare event. However, it is underreported. Patients with pulmonary endometriosis present with what is known as catamenial hemoptysis and/or catamenial pneumothorax. The first reported case of lung endometriosis was in 1938 [4]. The first case of catamenial pneumothorax due to endometriosis was reported in 1958 [5]. It is estimated that about 60% of pulmonary endometriosis cases are associated with pelvic endometriosis [6].

Catamenial hemoptysis is one of the manifestations of pulmonary endometriosis [7, 8]. It is of concern to the patient and the treating physician. Certainly, before the diagnosis is finalized one has to rule out other causes of hemoptysis in the form of lung infections and lung tumors.

Catamenial pneumothorax is another manifestation that is recurrent and occurs within 24 hours before the menses up to 72 hours after the onset of menstrual flow. It is estimated that about 1/3 of spontaneous pneumothorax presenting to hospitals is due to endometriosis [9].

The following case presentation is a patient with recurrent catamenial pneumothorax that was treated successfully with medical therapy.

## 2. Case Presentation

This is a 42-year-old married female patient who presented for consultation because of recurrent spontaneous catamenial right pneumothorax for the past four years. The patient is G2P2002. Her first pregnancy was the result of in vitro fertilization and the second pregnancy was spontaneous. She did not have any history of pelvic or abdominal pain.

These recurrent episodes of pneumothorax have been evaluated by lung specialists and no lesions were found. There were no associated symptoms of hemoptysis or cough. Chest radiological studies failed to show any lesions. The patient was offered medical treatment with either Danazol or GnRH agonist. The patient preferred Danazol treatment to avoid vasomotor symptoms related to GnRH use.

The patient was then treated medically using Danazol 400 mg am and 400 mg pm for six months. The menstrual flow stopped. Pneumothorax did not recur again during the treatment. Later, followup revealed that she was symptom free.

## 3. Discussion

Endometriosis is an enigma because its etiology is theories, its life history is not known, and its recurrence after treatment is relatively high. The first description of the disease by Rokitansky in 1956 was mainly directed towards the pathogenesis of endometriosis. Over the years it has been realized that endometriosis is a disease that spreads to various parts of the body including the various organs in the abdominal cavity and cesarean section scars. Recently, we started to receive reports on the category of pulmonary endometriosis with its manifestations in the form of catamenial hemoptysis or catamenial pneumothorax or both. Studies showed that the disease mainly affects the right side of the chest much more commonly than the left side of the chest.

Endometriosis of the pleura may result from spread of endometriotic tissue from the abdominal cavity through a defect in the diaphragm, with the cells gaining access to the pleura, the lung, or both. It may also be the result of hematogenous or lymphatic spread to the lungs and pleura. Endometriosis may invade the pleura directly from endometriosis nodule on the diaphragm.

The management of these cases has been, in the majority, directed towards surgical intervention with thoracotomy, bronchoscopy, and excision/destruction of the lesion or has been creating adhesions of the parietal pleura to block the pleural space. With the introduction of medications that treat pelvic endometriosis, some investigators started to use these medications for the treatment of lung endometriosis [10–12].

In the present case with catamenial pneumothorax and negative radiological studies, medical treatment using Danazol was effective in leading to a remission of the disease. Danazol leads to a condition of pseudomenopause by suppression of GNRH and suppression of gonadotropins [13]. The patient will be hypoestrogenic due to suppression of the ovarian steroidogenesis. The case showed marked improvement without any recurrence of the disease process. Therefore, medical treatment of pulmonary endometriosis is a varied type of approach and is as successful as surgery. The patient will be treated on an ambulatory basis and therefore, there will be no interference with daily activities.

The diagnosis of pleural endometriosis in this case was made by the symptomatology of chest pain due to spontaneous pneumothorax associated with the menstrual periods. That is why it is called catamenial pneumothorax. It has also been found that pulmonary endometriosis affects older women as compared to younger women who are affected with pelvic endometriosis. The use of CT scan or MRI of the lung might show the lesion. However, in many cases there is no defined lesion with the exception of the occurrence of pneumothorax. It is also estimated that about 50–80% of patients who have pulmonary endometriosis also have pelvic disease [4, 6, 14, 15]. This patient had no abdominal or pelvic symptoms to require any laparoscopic evaluation.
